# Elexacaftor-Tezacaftor-Ivacaftor Treatment Reduces Abdominal Symptoms in Cystic Fibrosis-Early results Obtained With the CF-Specific CFAbd-Score

**DOI:** 10.3389/fphar.2022.877118

**Published:** 2022-06-03

**Authors:** Jochen G. Mainz, Carlos Zagoya, Louise Polte, Lutz Naehrlich, Lenny Sasse, Olaf Eickmeier, Christina Smaczny, Anton Barucha, Lilith Bechinger, Franziska Duckstein, Ludwik Kurzidim, Patience Eschenhagen, Laura Caley, Daniel Peckham, Carsten Schwarz

**Affiliations:** ^1^ Cystic Fibrosis Center, Brandenburg Medical School (MHB) University, Klinikum Westbrandenburg, Brandenburg an der Havel, Germany; ^2^ Faculty of Health Sciences Joint Faculty of the Brandenburg University of Technology Cottbus-Senftenberg, The Brandenburg Medical School Theodor Fontane and the University of Potsdam, Potsdam, Germany; ^3^ Department of Pediatrics, Justus-Liebig-University Giessen, Giessen, Germany; ^4^ Universities of Giessen and Marburg Lung Center (UGMLC), German Center for Lung Research (DZL), Giessen, Germany; ^5^ Christiane Herzog CF-Zentrum Frankfurt am Main, Universitätsklinikum Frankfurt am Main CF-Zentrum, Frankfurt am Main, Germany; ^6^ CF-Zentrum Westbrandenburg, Campus Potsdam, Klinikum Westbrandenburg, Potsdam, Germany; ^7^ Leeds Institute of Medical Research at St James’s, University of Leeds, Leeds, United Kingdom; ^8^ Adult Cystic Fibrosis Unit, St James’s University Hospital, Leeds Teaching Hospitals NHS Trust, Leeds, United Kingdom

**Keywords:** gastrointestinal, patient reported outcome measure, CFTR modulators, elexacaftor, symptom score

## Abstract

**Background:** The novel and highly effective CFTR modulator combination of elexacaftor-tezacaftor-ivacaftor (ETI) has been shown to improve lung function and body weight in people with Cystic Fibrosis (pwCF) carrying a F508del mutation. However, the impact of these modulators on gastrointestinal (GI) symptoms is relatively unknown. Therefore, the CFAbd-Score was developed and validated following FDA recommendations for development of a PROM including focus groups, multidisciplinary CF specialists, people with CF and their families. The aim of this study was to assess effects of ETI on GI symptoms using the CFAbd-Score.

**Methods:** Gastrointestinal symptoms were prospectively assessed in pwCF using the CFAbd-Score before and up to 26 weeks during therapy. The CFAbd-Score was also administered to a healthy control (HC) group. The one-sided questionnaire includes 28 items grouped in five domains. Data analysis included calculation of scores with a weighting tool, developed according to FDA recommendations.

**Results:** A total of 107 pwCF attended in four CF centres in Germany and four centres in the UK completed the CFAbd-Score on at least two occasions. Results were compared to those obtained from the questionnaire of 45 HCs. Despite differences in demographics, age and proportion of pancreatic insufficiency between German and UK patients, analyses based on linear mixed-effects models at week 24 of ETI therapy revealed that estimated marginal means (EMMs) of total CFAbd-Scores significantly reduced (mean ± SE: 14.9 ± 1.2→10.6 ± 1.4; *p* < 0.01). Also EMMs of all five domains significantly declined (“pain” 16.3 ± 1.6→10.2 ± 2.3, “GERD” 15.8 ± 1.8→8.2 ± 1.9, “disorders of bowel movement” 20.9 ± 1.5→16.0 ± 1.7, “disorders of appetite” 7.9 ± 1.1→2.6 ± 1.1 and “quality of life impairment” 10.1 ± 1.92→3.9 ± 1.9). However, during 24 weeks, CF participants’ symptoms mostly still did not reach the reference levels of HCs.

**Discussion:** Using the CFAbd-Score, the first PROM specifically developed for assessment of CF-related abdominal symptoms, we demonstrate comprehensive improvements in GI symptoms after initiation of the highly effective modulator therapy ETI.

## 1 Introduction

Abdominal involvement is a hallmark of Cystic fibrosis (CF), the most common lethal inherited disease of the Caucasian population. The defective gene results in abnormalities in the production and function of the cystic fibrosis transmembrane conductance regulator (CFTR) protein (Riordan et al., 1989), an ATP gated anion channel which is highly expressed in both the respiratory and gastrointestinal systems (Ooi and Durie, 2016; Tipirneni and Woodworth, 2017; Bell et al., 2020). Lung disease has been the main focus for clinical and scientific research, as around 90% of people with CF (pwCF) die prematurely from respiratory failure. Dysfunctional ion transport in the airways results in dehydrated airway surface liquid, viscous mucus secretions, impaired muco-ciliary clearance and a predisposition to airway infections and heightened local and systemic inflammation. Although lung involvement remains the main cause of morbidity and mortality in pwCF, a multidisciplinary approach to treatment has significantly improved survival (Bell et al., 2020).

More recently, small molecules which can partially correct CFTR function have been introduced into clinical practice (Accurso et al., 2010). These highly effective CFTR-modulator therapies (HEMT) were initially available only for pwCF carrying rare gating mutations such as G551D, which is detected in about 1.3% of pwCF in Europe. Ivacaftor (IVA), the first CFTR-modulator approved for patients carrying a gating mutation, not only improved pwCF’s pulmonary function (percentage predicted FEV_1_ + 10%); it was also associated with a decrease of about 50% in sweat chloride concentrations. Furthermore, in pwCF receiving this CFTR modulator, general quality of life (QoL) and weight gain improved substantially. Some younger children with CF even recovered pancreatic sufficiency, and their previously lowered gastric pH became normal (Davies et al., 2016; Gelfond et al., 2017; Mainz et al., 2018).

A number of countries, including Germany and the UK, have recently approved a novel highly effective triple combination of two CFTR-correctors, tezacaftor and the new compound elexacaftor, together with the CFTR-potentiator ivacaftor for pwCF carrying the most frequent mutation F508del. This combination of elexacaftor/tezacaftor/ivacaftor (ETI) has since been approved for those with a least one F508del mutation from the age of 6 years and above.

The improvement in respiratory symptoms and life expectancy in pwCF has shifted focus towards better understanding of the non-pulmonary CF-related manifestations, especially GI involvement (Bell et al., 2020; Balfour-Lynn and King, 2020; J Burton et al., 2021). Consequently, abdominal symptoms resulting from loss or altered function of CFTR in the pancreas, gut and hepatobiliary system, impairing transport of chloride and sodium, bicarbonate and water, have recently come into scientific and clinical focus (Bodewes et al., 2015; Ooi and Durie, 2016; Freeman and Ooi, 2017; Bolia et al., 2018; Rowbotham et al., 2018). In 2016, a “James Lind Alliance Priority Setting Partnership in CF” conducted a systematic search for the top 10 research priorities in CF in hundreds of pwCF and healthcare providers. Answering the question “how can we relieve gastro-intestinal symptoms, such as stomach pain, bloating and nausea in people with cystic fibrosis” was the second highest priority question (Rowbotham et al., 2018).

Most trials regarding CFTR-modulators primarily focus on endpoints relating to pulmonary manifestations in pwCF. At the same time, there has been a lack of CF-specific patient reported outcome measures (PROMs) to assess abdominal symptoms and measure the impact of novel therapies on GI symptoms (Wainwright et al., 2015a; Rosenfeld et al., 2018; Middleton et al., 2019).

For this purpose, in 2017, at the time point when results of the above-mentioned survey were available, we published on the first CF-specific abdominal symptom score (Tabori et al., 2017a) developed following FDA Guidelines for development of a PROM (Food and Drug Administration, 2009). Elaborated in four published steps, the first version of this CF-specific questionnaire (initially named JenAbd-Score, and from then CFAbd-Score) aimed to assess and quantify abdominal involvement in pwCF, and its development included patients, proxies and health care specialists, as recommended by FDA guidelines (Tabori et al., 2017a; Tabori et al., 2017b; Jaudszus et al., 2019). At present, the CFAbd-Score is available in nine languages and implemented in many international studies (Boon et al., 2020; Mainz J et al., 2021; Jaudszus et al., 2021; Ng et al., 2021; Raun et al., 2022).

The aim of this study was to assess changes in abdominal symptoms with the CFAbd-Score during the first 24 weeks of a new highly effective CFTR-modulating ETI-therapy in pwCF from Germany and the UK. A secondary aim was to investigate whether there were differences in the magnitude of change of CFAbd-Score between those who were previously naïve to CFTR modulators and those who were on prior therapy. We also compared these results to healthy controls (HCs).

## 2 Methods

### 2.1 Participants

Participants were recruited from four CF care centres in Germany and four CF care centres in the United Kingdom. Inclusion criteria were: pwCF about to start ETI, age of 12 years or older (for the German cohort) or 18 years or older (for the UK cohort), a confirmed diagnosis of cystic fibrosis and the presence of F508del on at least one *CFTR* allele. For the German cohort, all eligible subjects were considered for the study regardless of their FEV_1_ values, comorbidities and multi-resistant or atypical organisms. For the UK cohort, those who were pancreatic sufficient, pregnant, had a prognosis of less than 6 months or had another significant GI pathology (such as short bowel syndrome, a colostomy or GI cancer) were excluded. Exclusion criteria for all participants were: inability to comply with the study procedures or assessments. For reference, a group of 45 healthy people from Germany was included as a control group, excluding people with chronic GI-diseases as well as food allergy or intolerance.

### 2.2 Assessment of Symptoms

Abdominal symptoms experienced by pwCF before and during therapy were captured with the CFAbd-Score, which in its final version consists of 28 items. Items refer to a recall period of 2 weeks and are grouped into five domains: pain (4 items), gastroesophageal reflux disease (GERD) (3 items), quality of life (QoL) impairment (8 items), disorders of bowel movement (DBM) (8 items) and disorders of appetite (DA) (5 items). Items also include a modified version of the Bristol Stool Scale. Local research coordinators administered prospectively copies of the CFAbd-Score questionnaire to participants at each study centre. The aim was for participants to complete the questionnaire before and up to 26 weeks after ETI therapy initiation at subsequent visit appointments. Questionnaires not collected within the 26-week time frame were not considered in the analysis. Scoring and analyses of completed questionnaires were centrally performed at the CF centre in Brandenburg an der Havel, Germany.

### 2.3 Statistical Analysis

In this study, linear mixed-effects models (LMEMs) for repeated measures allowed to compute estimated marginal means (EMM) for changes in the CFAbd-Score and its five domains at week 24, despite the unequal number of observations between subjects and the unequal time spacing between observation time-points (Supplementary Material). LMEMs are advanced statistical methods that allow dealing with missing data and/or imbalanced datasets without resorting to the use of artificial data-imputation methods (Laird and Ware, 1982). LMEMs approaches use all data points from each study participant and are superior to frameworks wherein all data points are simply pooled and average together regardless of their individual observation times.

Within this approach, a linear regression procedure fits an optimal straight line for each subject’s predicted and time-related variables, yielding intercepts and slopes that describe each line assigned to each subject. These coefficients are assumed to vary randomly according to the distribution of the whole cohort and, consequently, rely on within-subject information as well as the whole-cohort distribution’s characteristics. In this study, unless stated otherwise, 24-week means reported for the CFAbd-Score, its five domains, ppFEV_1_, BMI, BMI-for-age z-Scores (de Onis and Blössner, 2003) and weight are based on EMMs from the corresponding LMEM.

The LMEMs included time from therapy start (in weeks) as a fixed effect, and administration of previous CFTR modulators, age (as a binary variable, i.e., <18 vs. ≥ 18), sex and geographic location (as a binary variable, i.e., either Germany or UK) of the CF centre as factors. Global cohort changes in percent of predicted FEV_1_ (ppFEV_1_) were analysed with a mixed-effects model using time from therapy start (in weeks) as a fixed effect. Similar models were used to analyse body mass index (BMI) and weight in adults, as well as BMI-for-age z-Scores and weight in children. Student’s t-tests were performed to assess differences between scores from pwCF and healthy controls. All statistical analyses were performed using R version 3.6.3. Figures were created in Excel 2013, GraphPad Prism 8.4.3 (GraphPad Software Inc., La Jolla, CA, United States) and RStudio version 1.4.1717.

## 3 Results

A total of *n* = 107 pwCF attended in each of the four participating CF centres across Germany (Brandenburg an der Havel, Potsdam, Frankfurt and Giessen, *n* = 68 pwCF) and four UK CF centres (Leeds, Cambridge, Manchester and Birmingham, *n* = 39 pwCF) were included in this study. Prior to triple CFTR-modulator therapy, *n* = 60 patients had already been receiving a different CFTR-modulator therapy, whereas the remaining *n* = 47 were treatment naïve. More information about participants’ demographic and clinical characteristics is provided in Table 1. Overall, 252 questionnaires from pwCF were collected: 174 from Germany and 78 from the United Kingdom. Baseline questionnaires in the UK were collected with a mean of −294 ± 85 days [median, IQR: 296 (−255, −355) days; range (−449, −29) days] before ETI therapy, whereas in Germany the mean was −16 ± 28 days [median, IQR: 6 (−21, 0) days; range (−169, 0) days]. From baseline to the last completed questionnaire, participants had been on ETI therapy for a mean of 93 ± 41 days [median, IQR: 85 (71, 120) days; range (11, 179) days] and 130 ± 42 days [median, IQR: 134 (113, 159) days; range: range (23, 181) days] in the German and UK cohorts, respectively. During the 24-week time frame on ETI, one further questionnaire was administered to participants in the UK, whereas in Germany a mean of 2 ± 1 questionnaires per participant [median, IQR: 1, (1, 2) questionnaires per participant] were collected.

**TABLE 1:** Baseline demographics and clinical characteristics of the pwCF included in the study. None of the included pwCF carried a gating mutation, and none was previously treated with ivacaftor alone.

Participants	Whole cohort	Geographic region	
		Germany	United Kingdom
Total, n	107	68	39
Sex, female, n (%)act	55 (50.5)	39 (57.4)	16 (41.0)
Age, mean ± SD (years)	25.4 ± 10.9	21.0 ± 8.9	32.9 ± 9.9
median, IQR (years)	23 (18,32)	19 (14, 24)	30 (26,38)
range (years)	(12, 62)	(12, 55)	(19, 62)
<18, n (%)	26 (24.3)	26 (38.2)	0 (0)
≥18, n (%)	81 (75.7)	42 (61.8)	39 (100)
Genotype, n (%)			
F508del homozygous	68 (63.6)	40 (58.8)	28 (71.8)
F508del heterozygous	39 (36.4)	28 (41.2)	11 (28.2)
Previous CFTR modulator			
Yes, n (%)	60 (56.1)	34 (50.0)	26 (66.7)
Pancreatic function			
PS, n (%)	5 (4.7)	5 (7.4%)	0 (0)
PI, n (%)	102 (95.3)	63 (92.6)	39 (100)
Weight, mean ± SD (kg)	59.3 ± 13.6	54.6 ± 12.0	67.4 ± 12.3
Height, mean ± SD (cm)	166.3 ± 10.3	164.0 ± 9.9	170.2 ± 10.0

PS, pancreatic sufficient; PI, pancreatic insufficient.

Additionally, as a control group, 45 healthy controls from Germany reported on their abdominal symptoms over the past 2 weeks using the CFAbd-Score. Mean age resulted in 39.8 ± 22.8 years [median, IQR: 27 (26, 57) years; range: 10–84 years]; 48% were female. Healthy control group’s mean weight and height were 73.0 ± 23.1 kg and 167.0 ± 31.7 cm, respectively.

### 3.1 Changes in Total CFAbd-Score and its Domains

Prior to therapy initiation (mean ± SEM: 111 ± 13 days), 107 participants completed the CFAbd-Score, and, at least, once again within the first 26 weeks of ETI therapy.

**FIGURE 1:**
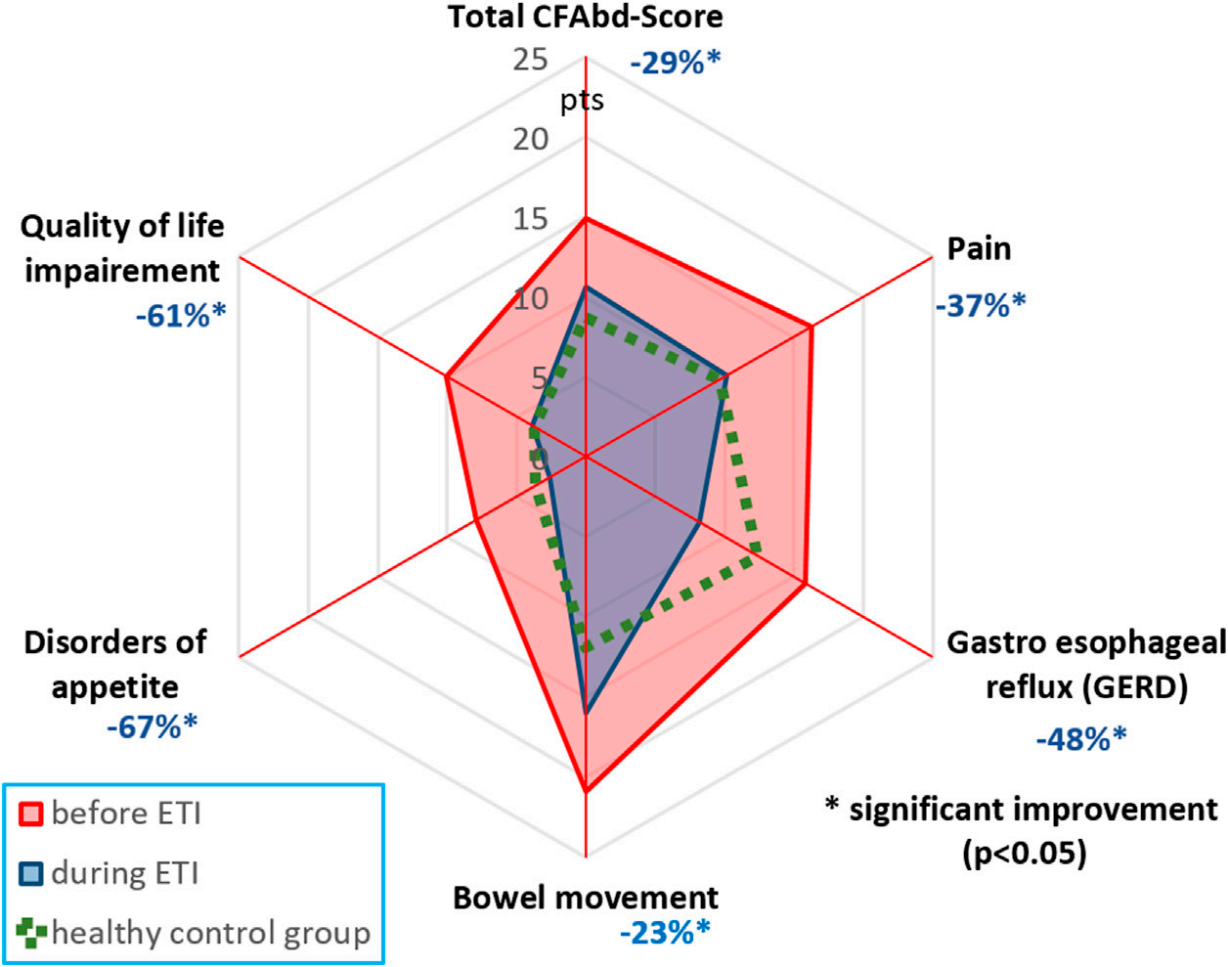
CFAbd-Score changes for the whole cohort and its 5 domains after therapy initiation (Table 1). Percent changes are calculated from estimated marginal means (EMMs) at week 24 of ETI therapy.

LMEM calculations at week 24 of ETI therapy, revealed that estimated marginal means of total CFAbd-Scores as well as mean scores for the pain, GERD, DBM, DA, and QoL impairment domains dropped significantly (*p* ≤ 0.01 for all) 0.7-fold, 0.7-fold, 0.5-fold, 0.8-fold, 0.4-fold, and 0.4-fold, respectively (Figure 1 and Table 2).

**TABLE 2:** Estimated marginal means of the total CFAbd-Score and its five domains at 24 weeks for the whole CF cohort (*n* = 107), as calculated according to the validated algorithm elaborated according to FDA guidelines for development of a PROM.

Domain	Baseline (points)	24 weeks (points)	Reduction factor	Healthy cohort
Total CFAbd-Score mean ± SE	14.9 ± 1.2	10.6 ± 1.4^∗∗^	0.7	8.7 ± 1.0^∗∗^
Pain mean ± SE	16.3 ± 1.6	10.2 ± 2.3^∗∗^	0.6	9.7 ± 2.1^∗∗^
GERD mean ± SE	15.8 ± 1.8	8.2 ± 1.9^**∗∗**^	0.5	12.4 ± 1.8 (n.s.)
DBM mean ± SE	20.9 ± 1.5	16.0 ± 1.7^∗∗^	0.8	11.9 ± 1.4^∗∗^
DA mean ± SE	7.9 ± 1.1	2.6±1.1^∗∗^	0.4	3.6 ± 1.0^∗∗^
QoL impairment mean ± SE	10.1 ± 1.92	3.9 ± 1.9^∗∗^	0.4	3.7 ± 1.1^∗∗^

∗∗: *p* < 0.01 for comparison with mean at baseline n. s.: not significant.

SE, standard error; GERD, gastroesophageal reflux disease; DBM, disorders of bowel movement; DA, disorders of appetite; QoL, quality of life.

### 3.2 Comparison of Abdominal Symptoms in pwCF Pre- and During ETI Treatment Compared to Reference Values From Healthy Controls

Overall, mean scores from pwCF at baseline were significantly higher (*p* < 0.01) than those from the healthy cohort, except for the GERD domain, where there was no statistically significant difference (Table 2).

For pwCF during ETI treatment, there were no significant differences in the total CFAbd-Score and four domains (pain, GERD, DA and QoL) when compared to healthy controls. The mean score for DBM domain remained however, significantly higher in the CF cohort during-ETI treatment compared to HCs (pwCF: 17.6 ± 1.3, HC: 11.9 ± 1.4; *p* = 0.004).

### 3.3 Differences Between Patient Subgroups

Pain domain scores for pwCF were higher in females than in males at baseline and after 24 weeks during ETI therapy (baseline: 1.6-fold; 24 weeks: 2.2-fold; both *p* = 0.003). Also in the GERD domain, female participants reported higher scores than males at baseline (1.9-fold, *p* = 0.012). However, the difference between these subgroups decreased during ETI therapy and was no longer significant after 24 weeks (1.2-fold; *p* > 0.1). Being on a prior CFTR modulator, which in the included patients only regarded lumacaftor + ivacaftor or tezacaftor + ivacaftor, had no impact on GI symptom burden at baseline or during ETI treatment when compared to drug naïve individuals. There was also no difference in total CFAbd-Scores between different sexes, genotypes (F508del homozygous vs. heterozygous) or age groups (<18 vs. ≥ 18).

However, there were marked differences between the UK and German cohorts, with the UK subgroup reporting higher overall total CFAbd-Scores than pwCF from Germany (baseline: 1.2-fold, *p* = 0.77). Following ETI therapy initiation, the German cohort (*n* = 68) reached a highly significant 0.4-fold decline (*p* < 0.001) in total CFAbd-Scores, in contrast to the UK cohort (*n* = 39), where a 0.9-fold declined did not reach significance.

Both groups showed similar levels of pain at baseline, but during ETI therapy pain levels declined sharply in the German cohort (*n* = 68; 0.2-fold, *p* < 0.001), whereas no decline was observed in the UK subgroup. There were no significant differences in the DBM domain, between UK and German cohorts at baseline. However, ETI therapy resulted in a 0.5-fold decline (*p* < 0.0001) in the German cohort, whereas, the mean score remained almost unchanged in the UK cohort (baseline: 23.6 ± 2.5, week-24: 23.5 ± 2.5; *p* > 0.1).

The QoL impairment domain markedly decreased in the German subgroup with ETI therapy (0.1-fold, *p* < 0.001) and decreased non-significantly in the UK cohort (0.9-fold with respect to baseline, *p* > 0.1). In the DA domain, there were no significant differences between the subgroups regarding previous CFTR-modulation therapy, sex, geographic region, genotype or age.

### 3.4 Exploratory Analysis of Prominent Gastrointestinal Symptoms and Their Impact on Quality of Life

**FIGURE 2:**
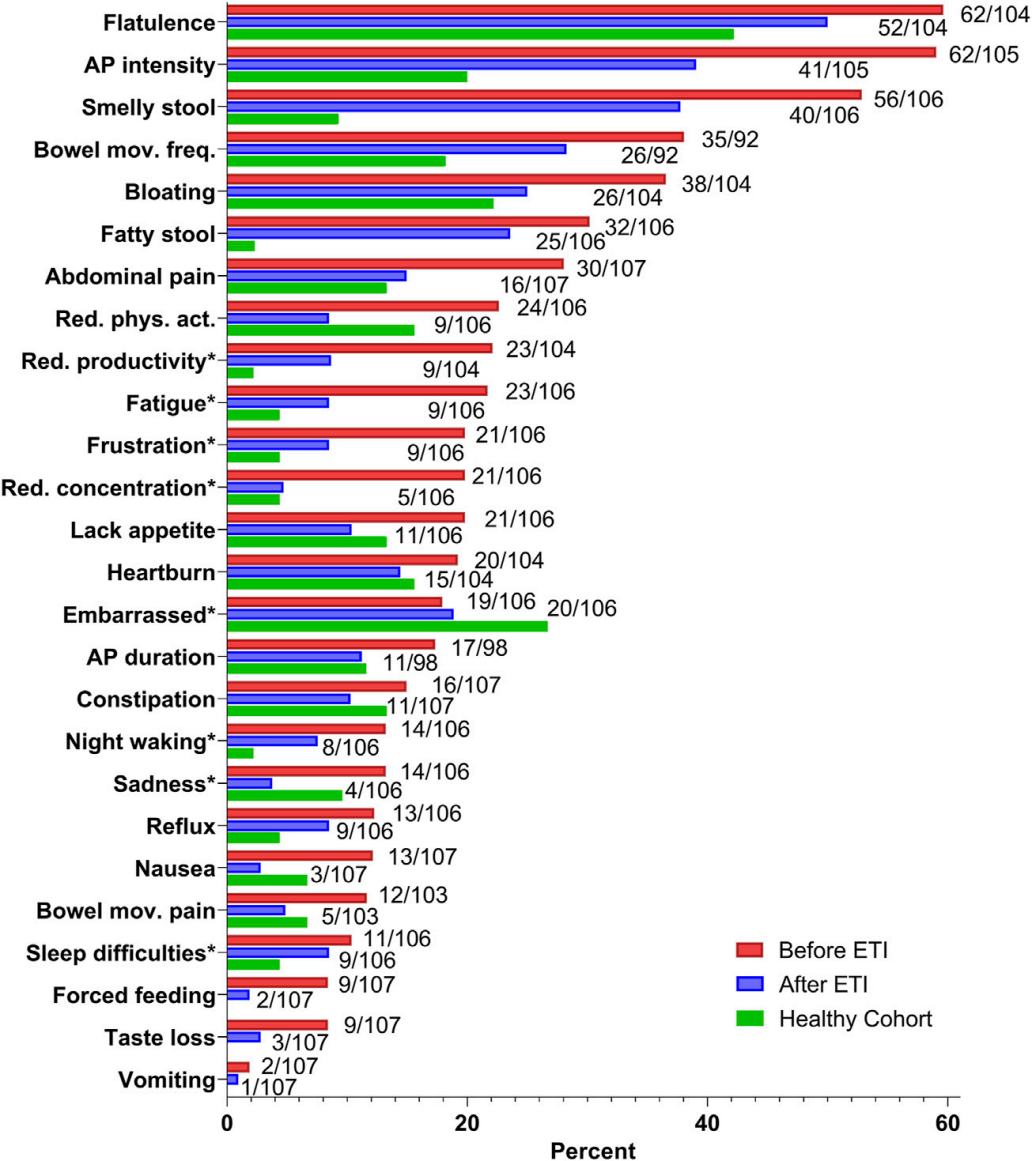
Changes in responses for single items included in the CFAbd-Score from 107 pwCF during treatment with Elexacaftor-Tezacaftor-Ivacaftor, compared to responses obtained from a cohort of 45 German healthy controls without food allergy or intolerance. Percentages of patients reporting each symptom at least occasionally (2–3 times during the past 2 weeks) are listed. Items displaying a total number of pwCF less than 107 indicate that a fraction of participants left some questions unanswered. The percentage of total unanswered questions over the whole study was 1.5%. From a total of 28 items included in the CFAbd-Score, stool colour and stool consistency, as parts of the modified Bristol stool scale, were excluded. For frequency of bowel movement, changes regard those reporting at least 2–3 stools/day. *Items regarding quality of life were assessed as relating to abdominal symptoms. AP: abdominal pain; Bowel mov. freq.: bowel movement frequency; Red. phys.act.: Reduced physical activity.

Exploratory analyses comparing the percentage of participants self-reporting on symptoms experienced at least “occasionally” (2–3 times during the past 2 weeks) revealed flatulence as the most frequent symptom before ETI initiation (Figure 2). During ETI therapy, however, reports related to this question decreased from 60 to 50%. Abdominal pain (AP) intensity was the second most frequent symptom, decreasing from 59 to 39%. In regard to smelly stools, fatty stools and frequency of bowel movements, we found a marked improvement during ETI. However, the prevalence of these reported symptoms remained higher in the CF cohort compared to the HC.

A marked improvement was also seen for the majority of QoL symptoms related to abdominal symptoms in pwCF. At baseline, more pwCF reported that their GI symptoms impacted on physical activity, compared to HCs. However, following ETI therapy initiation, this was reduced to levels lower than those reported by HCs.

**FIGURE 3:**
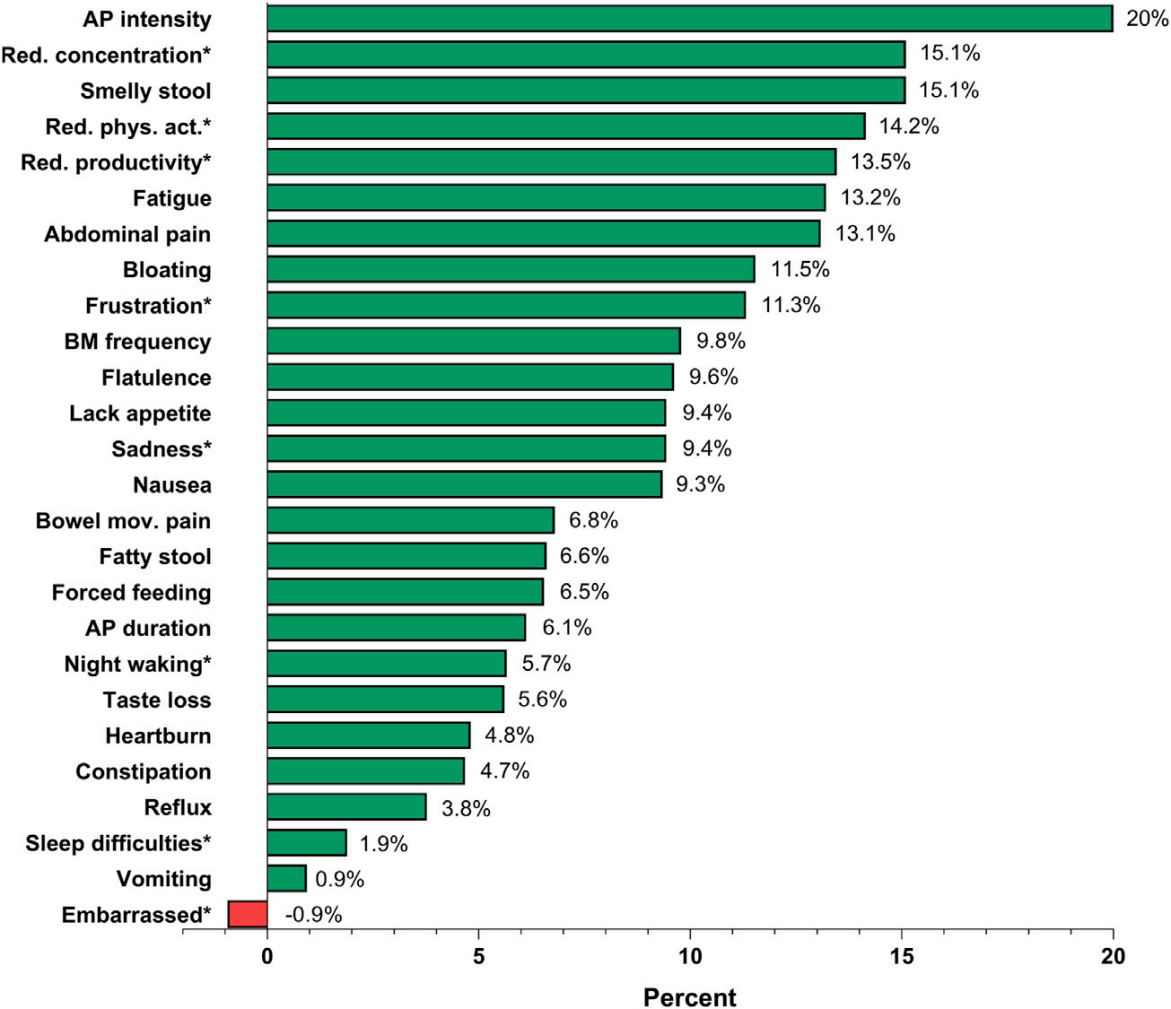
Pre/Post-ETI differences in responses for single items included in the CFAbd-Score from 107 pwCF during treatment with Elexacaftor-Tezacaftor-Ivacaftor (ETI). Percentages of patients reporting each symptom at least occasionally (2–3 times during the past 2 weeks) are listed. Stool colour and stool consistency, as parts of the modified Bristol stool scale, were excluded. For frequency of bowel movement, changes regard those reporting at least 2–3 stools/day. AP: abdominal pain; Bowel mov: bowel movement; Red. phys.act: Reduced physical activity. *Items regarding quality of life were assessed as relating to abdominal symptoms.

Differences between pre- and during-ETI frequency responses for single CFAbd-Score’s items (Figure 3) revealed that abdominal pain intensity was the symptom that improved the most during ETI therapy (20%), followed by “reduced concentration” and “smelly stool” (15.1% for both). One of the two pwCF who reported suffering from vomiting at baseline experienced an improvement in this symptom during ETI therapy (Figure 2 and Figure 3).

### 3.5 Changes in Pulmonary Function, Body-Mass Index, and Weight in Children and Adults

**FIGURE 4:**
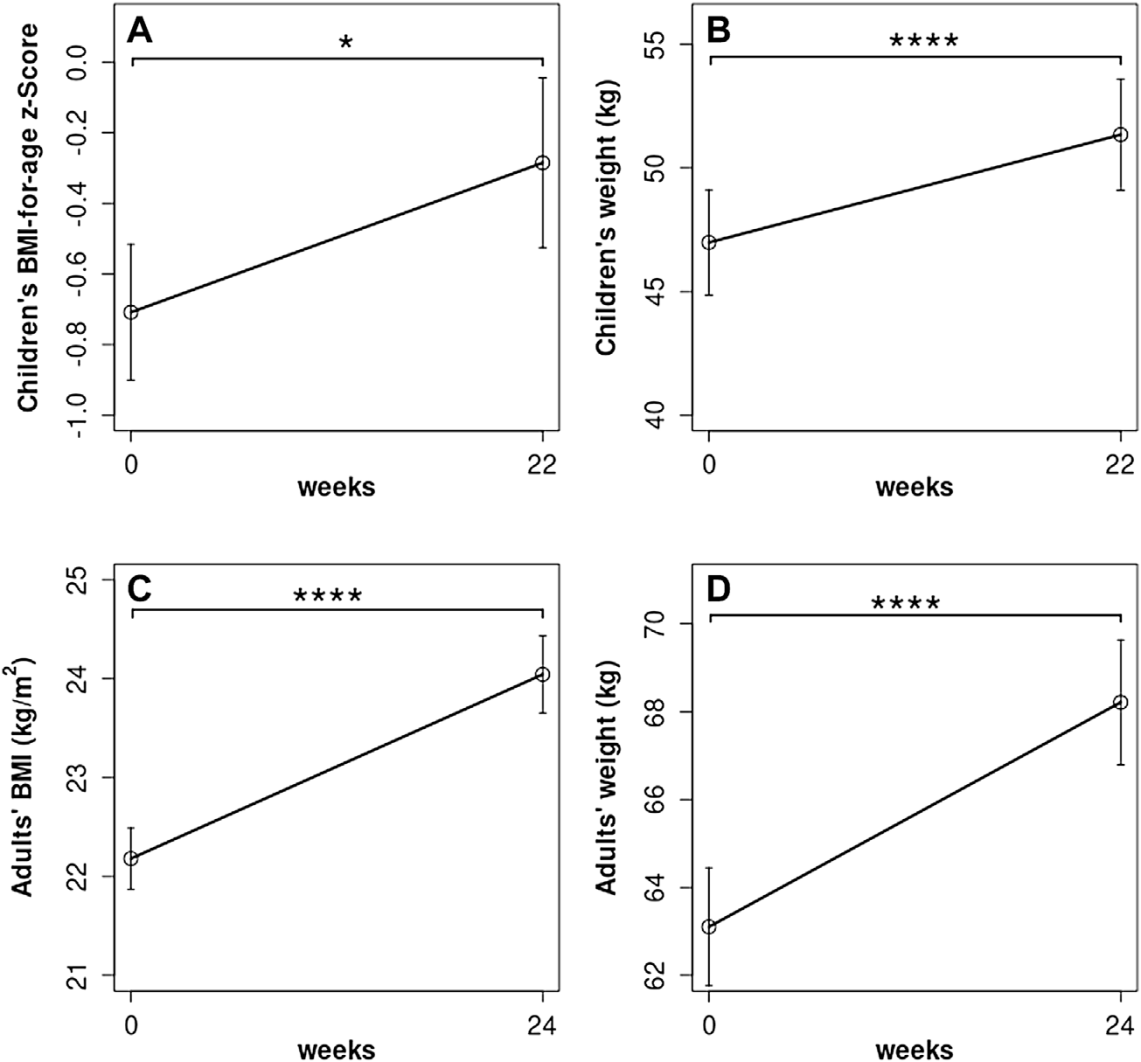
**(A)** Estimated marginal means (EMM) for children (*n* = 26) BMI-for-age z-scores and **(B)** weight during the ETI therapy. **(C)** Adult’s EMM BMI and **(D)** weight. **∗**: *p* < 0.05, ∗∗∗∗: *p* < 0.0001.

Pulmonary function assessed with ppFEV_1_ was observed to increase 13 percentage points (pp) during ETI therapy compared to baseline (baseline: 65.9 ± 2.5 pp, ETI: 79.4 ± 2.6 pp; *p* < 0.0001). Weight and BMI-for-age z-Scores in children were only available up to week 22 of ETI treatment, and, during this period of time, means for BMI-for-age z-Scores in children rose from −0.71 ± 0.19 to −0.29 ± 0.24 (*p* = 0.02) (Figure 4A). Within the same time span, mean weight in children increased from 47.0 ± 2.1 kg to 51.4 ± 2.3 kg (*p* < 0.0001) (Figure 4B). In adults, mean BMI increased by 8% from baseline compared to under ETI treatment (baseline: 22.2 ± 0.3 kg/m^2^; ETI: 24.0 ± 0.4 kg/m^2^; *p* < 0.0001), and mean weight was observed to increase by 8% (baseline: 63.1 ± 1.3 kg; ETI: 68.2 ± 1.4 kg; *p* < 0.0001) (Figures 4C,D).

## 4 Discussion

In this prospective study of 107 pwCF attended eight CF care centres across Germany and the UK, we quantified abdominal symptoms in pwCF before and during ETI therapy with the CFAbd-Score. This is presently the only CF-specific score for abdominal symptoms established and validated following FDA guidelines for development of a PROM. As part of validation, we developed an algorithm, which weights each item and domain included in our PROM differently, in order to gain optimal insights into abdominal symptoms in pwCF: a maximum of 100 points can be achieved, with higher scores accounting for higher burden of symptoms (Food and Drug Administration, 2009; Tabori et al., 2017a; Tabori et al., 2017b; Jaudszus et al., 2019; Boon et al., 2020; Jaudszus et al., 2021; Ng et al., 2021; Raun et al., 2022).

Our results show that ETI therapy has a highly significant direct impact on self-reported abdominal symptoms in pwCF. We found that the mean total CFAbd-Score decreased considerably by 29% during ETI therapy (14.9 ± 1.2 to 10.6 ± 1.4 pts, *p* = 0.0001). Similarly, mean scores for all 5 domains were observed to decrease significantly during the observation time frame, indicating a substantial reduction of different GI symptoms in the participants (Figure 1). Highest declines were seen for the domain “Disorders of appetite” (−67%), followed by “GI-related QoL impairment” (−61%), GERD (−48%), “Pain” (−37%), and “Disorders of bowel movement” (−23%) (all *p* < 0.01). Remarkably, in pwCF treated with ETI, the burden of estimated symptoms went down to levels approximating those from healthy controls in 4 of 5 domains, assessed with our CF-specific PROM, except for “Disorders of bowel movement” (Heijerman et al., 2019; Middleton et al., 2019). Remarkably, these findings reflect adverse events reported in a phase three study of elexacaftor-tezacaftor-ivacaftor, where 12.9% (26 of 202) of pwCF reported “Diarrhea” as a symptom, versus 7% (14 of 201) reporting disorder of bowel movement during placebo treatment (Middleton et al., 2019). However, as this trial did not include a validated CF-specific score for abdominal symptoms, we cannot estimate the entire scope and the dynamics of abdominal symptoms in both treatment arms.

Comparison of changes in domains during therapy with ETI in pwCF to averages of CFAbd-Scores obtained from healthy controls showed a trend towards normalization during HEMT in calculated total scores, regarding “pain”, “disorders of bowel movement” and “GI-related QoL”, without fully reaching levels of HC during early phases of ETI therapy. Remarkably, during therapy, pwCF reported even lower levels of “disorders of appetite” and lower “GERD-symptoms” than the healthy control group. It should be taken into account, that PROMs are subjective reports. Accordingly, it would be interesting for further studies to combine endpoints like ph-metry, in order to objectively assess rates of reflux and its improvement during HEMT. Our data obtained with the CF specific PROM accounts for the patients’ relief of the assessed symptom burden from a subjective perspective different for each individual.

Levels of single GI symptoms in pwCF, before ETI treatment initiation, were similar to those described in our previous studies, with predominant symptoms: pain, flatulence, smelly stools and frequency of bowel movements (Figure 2) (Tabori et al., 2017a; Jaudszus et al., 2019; Jaudszus et al., 2021). After ETI treatment, “Embarrassment due to GI symptoms” was increased but remained lower than those described by HCs. This could be explained by higher expectations following a general clinical improvement during the new therapy.

Interestingly, in our cohort, the reported reduction in abdominal symptoms was independent from prior CFTR modulator therapy of lumacaftor + ivacaftor or tezacaftor + ivacaftor, the first two approved dual combinations of a CFTR-corrector and potentiator (none of the included patients had received ivacaftor alone or a previous treatment with ETI). This is consistent with previous findings of comparatively low effects of these double modulators on pulmonary function, weight gain, reduction in sweat chloride concentrations and on other endpoints (Wainwright et al., 2015b; Elborn et al., 2016; Heijerman et al., 2019).

In parallel to the improvement in CFAbd-Scores, ppFEV_1_ increased by 13% and BMI and weight in adults increased by 8 and 9%, respectively, with ETI, as might be expected from previous studies (Heijerman et al., 2019; Middleton et al., 2019; Sutharsan et al., 2021). Significant improvements in children’s BMI-for age z-scores and weight were also observed during a 22-week follow-up. Again, this is consistent with reports from phase three trials with ETI (Heijerman et al., 2019; Middleton et al., 2019).

To our knowledge, this is the first publication assessing gastrointestinal symptoms with a validated CF-specific PROM, in pwCF receiving ETI therapy (Ramsey et al., 2011; Davies et al., 2013; Heijerman et al., 2019; Middleton et al., 2019). The markedly positive results, regarding a reduction of abdominal symptoms during the highly effective modulator therapy accord to previously described effects of HEMT on pathological processes in the digestive system of pwCF. In younger children carrying a G551D gating mutation, ivacaftor was shown to improve residual exocrine pancreatic function, as seen by weight gain and increased pancreatic elastase in stool (Rosenfeld et al., 2018). Some patients have even become pancreatic sufficient with ivacaftor therapy (Mainz et al., 2018; Rosenfeld et al., 2018; Mainz J. G et al., 2021). Such marked effects are not expected in pwCF carrying two severe non-gating mutations, such as F508del, owing to their extent of pancreatic destruction present in early life. However, a recent case report showed that a female with CF treated with ETI during pregnancy, delivered a F508del homozygous child with initial pancreatic function within the normal range and false-negative CF newborn screening (Fortner et al., 2021). Further studies suggest substantial improvement in other abdominal organs in pwCF receiving potent CFTR modulator therapies. This includes improvement of gastric hyperacidity after 1 month of treatment with ivacaftor (Gelfond et al., 2017). Furthermore, UK and US CF-registry data over 5 years reveal trends for lower incidences of CF-related diabetes in patients treated with ivacaftor (Volkova et al., 2020).

### 4.1 Limitations

Marked differences were found between the German and UK cohorts. While the decline in the total and domain scores from the German cohort was significant, this trend was markedly weaker in the UK cohort. We attribute these discrepancies to the following factors:

1) German CF centres included pwCF aged 12 years or over (mean ± SD: 21.0 ± 8.9), whereas the United Kingdom centres only included adults aged 18 years or above (mean age ±SD: 32.9 ± 9.9). Potentially, by adulthood, chronic inflammation and structure tissue damage may result in GI symptomology being less amenable to change. Furthermore, the United Kingdom cohort comprised only PI individuals whereas five German patients were pancreatic sufficient (PS). Interestingly, in our previous studies for development and validation following FDA guidelines for development of a PROM, in a cohort of 116 pwCF, we found that PI patients reported more frequently on diarrhoea, foul-smelling and fatty stools than PS individuals (*n* = 9) (Jaudszus et al., 2019). Furthermore, in this cohort, patients with a milder class IV–VI mutation on the second allele (*n* = 16) reported more often on abdominal pain than patients with two severe mutations. This could partly be attributed to pancreatitis, as mainly occurs in PS patients with remaining functional pancreatic-tissue. However, as in the present cohort only 4.7% resulted to be PS, differences between PI and PS did not reach significance. This relatively low proportion of PS patients, compared to 12–15% of PS patients reported in international registry data, are due to the fact that ETI is approved solely for pwCF carrying an F508del mutation, which many of the PS patients do not carry. A further reason for differences between the cohorts included in Germany and the United Kingdom may be attributed to dietary differences between the two cohorts which may exacerbate or reduce GI symptomology. Comparing changes in diets during a new highly effective CFTR-modulating therapy would be a highly interesting focus for future trials implementing a CF-specific validated PROM like the CFAbd-Score.
2) The study was performed during the intercurrent COVID-19 pandemic. Especially in the United Kingdom centres, this resulted in a delay between the administration of the CFAbd-Score post-commencing ETI therapy. As mentioned above, there were different data collection time frames. Baseline questionnaires in the United Kingdom were collected with a mean of -294 ± 85 days [median, IQR: 296 (−255, −355) days] before ETI initiation, whereas in Germany this time frame was −16 ± 28 days [median, IQR: 6 (−22, 0) days]. In addition, from baseline to the last completed questionnaire, participants had been on ETI therapy for a mean of 93 ± 41 days [median, IQR: 85 (71, 120) days] and 130 ± 42 days [median, IQR: 134 (113, 159) days] in the German and United Kingdom cohorts, respectively. In consecutive studies, repeated assessment of the CFAbd-Score at different time points of therapy with ETI would be of high interest, in order to observe the dynamics over longer time periods.
3) In regard to availability of our PROM in different languages, we must take into account that its translation always bears a certain risk of differences in the meaning of passages and/or words, as well as cultural differences, e.g., the concept of embarrassment between countries. Furthermore, it would have been valuable to have also compared our findings to a United Kingdom HC cohort. However, language differences as a major source of bias can be ruled out, as the English version of the CFAbd-Score has also been implemented in a parallel project in the RECOVER study, which we recently presented at the NACFC 2021 as a pilot-study poster and published in JCF as an abstract. In this study, performed in Ireland and the United Kingdom, the CFAbd-Score is being implemented for assessment of ETI effects in a wide range of organs involved in CF (Mainz J et al., 2021). At the NACFC, we presented preliminary results from such a study with a cohort of 60 pwCF, observed shortly before and 1 month after ETI initiation. Levels of significant improvement of CFAbd-Scores resulted similar to those observed at 24 weeks in the present cohort. The final results from the RECOVER project are planned to be published as longitudinal data on abdominal symptoms (CFAbd-Score) together with changes in markers of gut inflammation and pancreatic status over a total of 2 years of therapy. Given the higher levels of symptom burden observed in the United Kingdom cohort after a longer time of ETI administration, it is important to further investigate whether the significant reduction in GI symptoms seen after 24 weeks of ETI are sustained or attenuate with time and the impact of other intervening factors including COVID-19.

### 4.2 Validation of the CFAbd-Score's Sensitivity to Changes Following FDA Guidelines for Development of a Patient Reported Outcome Measures

During development of our CF-specific abdominal score, we meticulously complied with recommendations provided by the FDA for development of a PROM (Tabori et al., 2017a; Tabori et al., 2017b; Jaudszus et al., 2019; Jaudszus et al., 2021). We repeatedly included focus groups with professionals from different fields of CF care, pwCF and their families/proxies, in order to build up a questionnaire that includes the most relevant fields and questions, weighting results optimally. We found that the final version of the PROM including 28 items grouped into 5 domains has a very high degree of validity and reliability (Jaudszus et al., 2019). Comparing results from more than 100 pwCF with those from more than 80 healthy controls with the same tool, assessing “known-groups validity”, resulted in detection of markedly higher scores in pwCF, which is consistent with our findings presented in this publication (Jaudszus et al., 2019). Thereby, we found CFAbd-Scores to be higher in pwCF with a history of abdominal surgery (e.g., for meconium ileus and DIOS) as well as in pwCF with exocrine pancreatic insufficiency (Tabori et al., 2017a; Tabori et al., 2017b). Also, in preceding studies, CFAbd-Scores resulted to be higher in patients with abnormalities in pancreatic ultrasound: pancreatic lipomatosis was significantly correlated to a higher burden of GI-symptoms (*p* = 0.036) (Tabori et al., 2017b). More recently, we assessed intestinal inflammation in stool by faecal elastase, M2-pyruvate kinase (M2-PK), interleukins IL-1β, IL-6, IL-8, and neutrophil elastase (NE), together with the CFAbd-Score. Here we found that both, CFAbd-Scores and intestinal inflammation, most sensitively assessed by faecal elastase, are markedly higher in pwCF, compared to healthy controls (Jaudszus et al., 2021).

Besides assessment of ETI effects on abdominal symptoms, the present study also contributes to the final validation step of the CFAbd-Score: assessment of sensitivity to changes, defined as a statistically significant change after treatment. Accordingly, significant changes in GI symptoms before and during ETI captured at baseline and over 24 weeks with the CFAbd-Score proves, indeed, that the score possesses high sensitivity to changes and, therefore, represents a valuable tool in clinical routine settings.

Additionally, the CFAbd-Score has been implemented in various international studies, including studies relating to functional abdominal MRI (Ng et al., 2021). The CFAbd-Score has been translated into nine languages including Italian, Spanish, Portuguese, French, Dutch, Flemish and Danish, and therefore, it has been included in an international European multicentre trial (MyCyFapp) in order to assess abdominal symptoms during a project for optimization of PERT supplementation (Boon et al., 2020). Also a Danish group from the Copenhagen CF Centre assessed optimal timing of PERT, with inclusion of the CFAbd-Score (Raun et al., 2022). Besides these international projects, our PROM is presently implemented in a series of academic and industry studies.

At this historic point in time, we had the opportunity to investigate changes during a new highly effective CFTR-modulator combination, which in the CF community is seen as a new era in therapy for pwCF carrying a F508del mutation (Heijerman et al., 2019; Middleton et al., 2019), the most frequent CFTR-mutation in Europe, UK, US, and Australia. Accordingly, in contrast to the previous highly effective CFTR-modulator ivacaftor, available only for around 1.6% of pwCF in Europe, we now encounter availability of highly effective modulators for the majority of our patients (Naehrlich et al., 2019).

### 4.3 Conclusion

To our knowledge, this is the first study assessing gastrointestinal symptoms, with a validated CF-specific PROM, in pwCF receiving a CFTR modulator therapy. We demonstrate that therapy with ETI results in a substantial reduction of abdominal symptoms, independent from previous, less effective, treatment with CFTR modulators. Our CF-specific instrument reveals to be highly sensitive for capturing changes during a potent therapy, making the CFAbd-Score a valuable tool for implementation in interventional and observational studies, as well as for use in clinical routine.

## Data Availability

The raw data supporting the conclusions of this article will be made available by the authors, without undue reservation.
