# Corrigendum: Elexacaftor-Tezacaftor-Ivacaftor treatment reduces abdominal symptoms in cystic fibrosis-early results obtained with the CF-specific CFAbd-Score

**DOI:** 10.3389/fphar.2023.1207356

**Published:** 2023-05-03

**Authors:** Jochen G. Mainz, Carlos Zagoya, Louise Polte, Lutz Naehrlich, Lenny Sasse, Olaf Eickmeier, Christina Smaczny, Anton Barucha, Lilith Bechinger, Franziska Duckstein, Ludwik Kurzidim, Patience Eschenhagen, Laura Caley, Daniel Peckham, Carsten Schwarz

**Affiliations:** ^1^ Cystic Fibrosis Center, Brandenburg Medical School (MHB) University, Klinikum Westbrandenburg, Brandenburg an der Havel, Germany; ^2^ Faculty of Health Sciences Joint Faculty of the Brandenburg University of Technology Cottbus-Senftenberg, The Brandenburg Medical School Theodor Fontane and the University of Potsdam, Potsdam, Germany; ^3^ Department of Pediatrics, Justus-Liebig-University Giessen, Giessen, Germany; ^4^ Universities of Giessen and Marburg Lung Center (UGMLC), German Center for Lung Research (DZL), Giessen, Germany; ^5^ Christiane Herzog CF-Zentrum Frankfurt am Main, Universitätsklinikum Frankfurt am Main CF-Zentrum, Frankfurt am Main, Germany; ^6^ CF-Zentrum Westbrandenburg, Campus Potsdam, Klinikum Westbrandenburg, Potsdam, Germany; ^7^ Leeds Institute of Medical Research at St James’s, University of Leeds, Leeds, United Kingdom; ^8^ Adult Cystic Fibrosis Unit, St James’s University Hospital, Leeds Teaching Hospitals NHS Trust, Leeds, United Kingdom

**Keywords:** gastrointestinal, patient reported outcome measure, CFTR modulators, elexacaftor, symptom score

In the published article, there was an error in Figure 1 as published. The figure itself including the proportion of changes is correct. However, the numbers representing percent changes in reduction of symptoms do not correspond to those mentioned in the text but rather to a preliminary calculation from a smaller cohort of patients. Noteworthy, the improvement in abdominal symptoms assessed with the CFAbd-Score during ETI is in fact markedly higher in the now corrected version. The corrected Figure 1 and its caption appear below.

**FIGURE 1:**
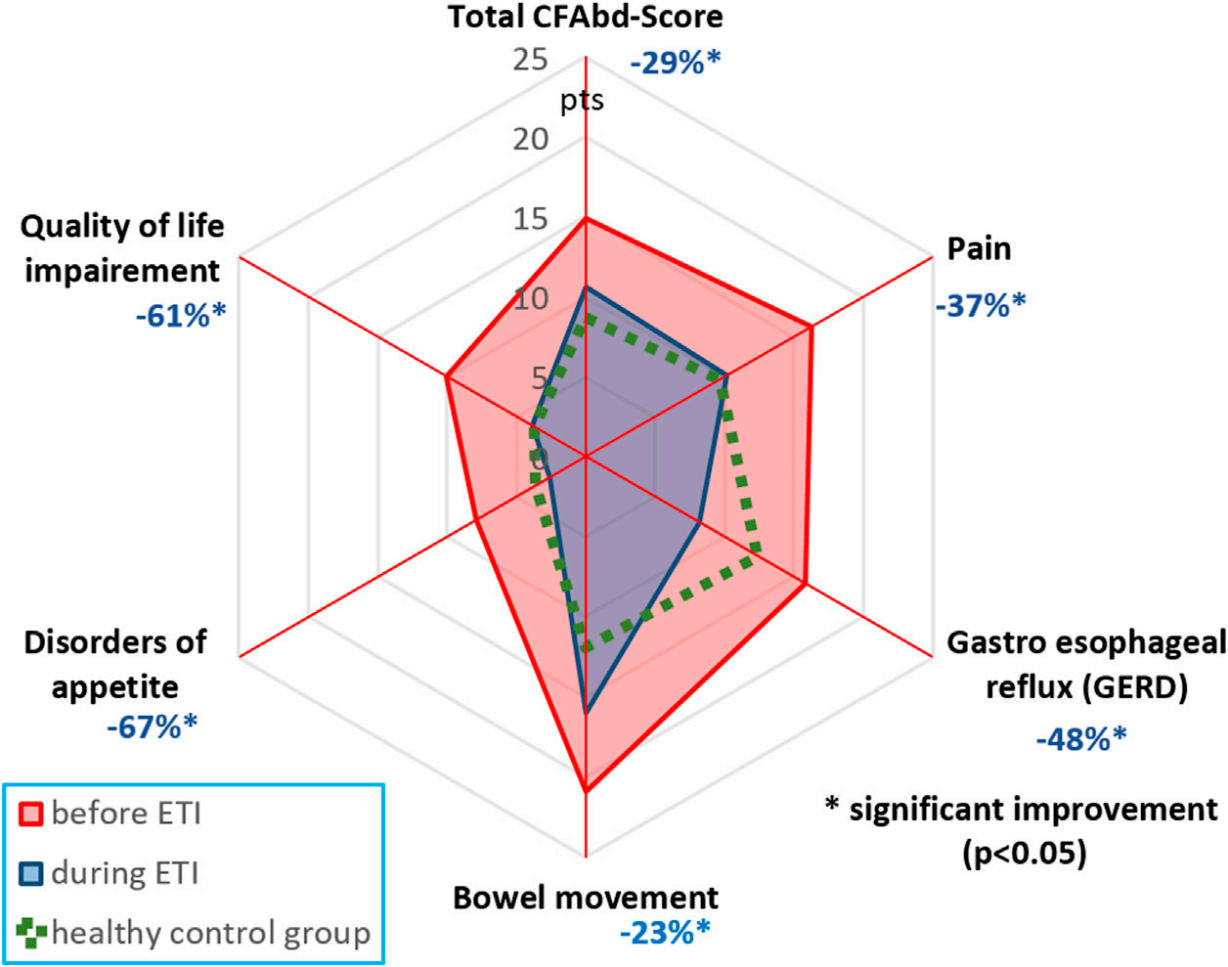
CFAbd-Score changes for the whole cohort and its 5 domains after therapy initiation (**Table 1**). Percent changes are calculated from estimated marginal means (EMMs) at week 24 of ETI therapy.

The authors apologize for this error and state that this does not change the scientific conclusions of the article in any way. The original article has been updated.

