# Supplementary material for: Elexacaftor-Tezacaftor-Ivacaftor Treatment Reduces Abdominal Symptoms in Cystic Fibrosis-Early results Obtained With the CF-Specific CFAbd-Score
Source: Front Pharmacol. 2022 Jun 3;13:877118. doi: 10.3389/fphar.2022.877118 (PMC9203829; doi:10.3389/fphar.2022.877118)
Supplement: Supplementary file 1 [file DataSheet1.docx]

***Supplementary Material***

1. **Statistical analyses – linear mixed-effects models**

Given the imbalanced and inhomogeneous data distribution resulting from this study, linear mixed-effects models (LMEMs) for repeated measures were used to compute estimated marginal means (EMM) for the CFAbd-Score and its five domains at week 24. LMEMs are advanced statistical methods that allow dealing with missing data and/or imbalanced datasets without resorting to the use of artificial data-imputation methods (27). Within this approach, a linear regression procedure fits an optimal straight line for each subject’s predicted and time-related variables, yielding intercepts and slopes that describe each line assigned to each subject (**Figure S1**).


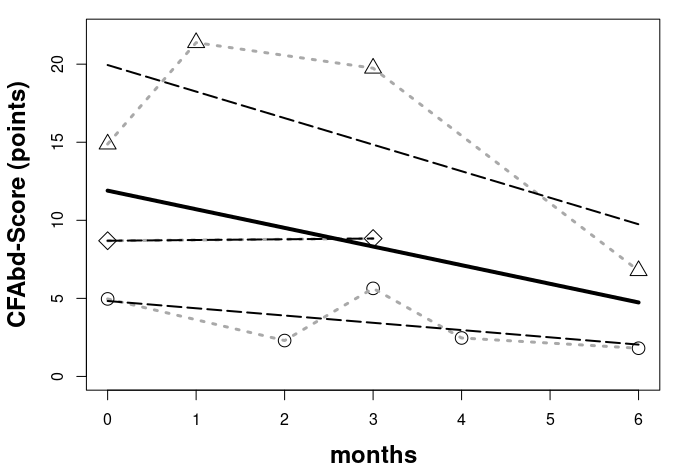


**Figure S2.** Three selected people’s data points. Symbols (triangles, diamonds, and circles) connected by dotted lines (dotted lines in gray) represent CFAbd-Scores at a specific time from a specific individual. Regression lines that fit the best each individual’s data points are represented by dashed lines in black, whereas the overall regression line is depicted as the thicker solid line.

Those coefficients are assumed to vary randomly according to the whole cohort’s distribution and, consequently, rely on within-subject information as well as on the whole-cohort distribution’s characteristics. In addition to the assumed random effects, fixed effects are usually included into the model, i.e. a fixed coefficient accounting for the overall trend of the data points.
When the number of data points during the observation period differs for each individual, the estimated coefficients as well as other estimations for each subject are based not only on single-subject data, but also on the whole cohort distribution. Therefore, this framework allows performing estimations for estimated means despite missing values, imbalanced data and differences in the number of measure per individual.

**References**

1. Laird NM, Ware JH. Random-effects models for longitudinal data. Biometrics. 1982;38(4):963-74.
